# Tranexamic acid reduces hidden blood loss in the treatment of intertrochanteric fractures with PFNA: a single-center randomized controlled trial

**DOI:** 10.1186/s13018-017-0625-9

**Published:** 2017-08-15

**Authors:** Jinlai Lei, Binfei Zhang, Yuxuan Cong, Yan Zhuang, Xing Wei, Yahui Fu, Wei Wei, Pengfei Wang, Shiming Wen, Hai Huang, Hu Wang, Shuang Han, Shuguang Liu, Kun Zhang

**Affiliations:** Department of Orthopaedic Trauma, Xi’an Honghui Hospital, Xi’an Jiaotong University Health Science Center, No 555, Youyi East Road, Xi’an, Shaanxi Province China

**Keywords:** Tranexamic acid, Hidden blood loss, Intertrochanteric femora, Fracture, PFNA, Randomized controlled trial

## Abstract

**Background:**

Hidden blood loss is a major concern for patients undergoing hip surgery for intertrochanteric fracture. The objective of this study was to investigate whether tranexamic acid (TXA) could reduce postoperative hidden blood loss in patients undergoing hip surgery for intertrochanteric fracture.

**Methods:**

A total of 77 patients with intertrochanteric fracture were enrolled in this randomized controlled study. Patients received either 200 mL (1 g) of TXA (*n* = 37) or normal-saline (NS) (*n* = 40) i.v. before hip surgery using proximal femoral nail anti-rotation (PFNA). Hemoglobin and hematocrit levels were measured preoperatively and postoperatively at day 1 and 3. Visible and hidden blood loss volumes were calculated at postoperative day 3.

**Results:**

On postoperative day 3, the transfusion rate was significantly lower in the TXA group compared to the NS group, although mean hemoglobin and hematocrit levels were not significantly different between the two groups. However, the estimated hidden blood loss volume (210.09 ± 202.14 mL vs. 359.35 ± 290.12 mL; *P* < 0.05) and total blood loss volume (279.35 ± 209.11 mL vs. 417.89 ± 289.56 mL; *P* < 0.05) were significantly less in the TXA group compared to the NS group, respectively.

**Conclusion:**

TXA significantly reduced postoperative hidden blood loss in patients with intertrochanteric fracture who underwent PFNA.

(Registration number: ChiCTR-INR-16008134).

## Background

Globally, hip fracture is a frequent cause of morbidity and mortality, especially in elderly people [1, 2]. Intertrochanteric fracture is one of the three major types of hip fracture, comprising approximately half of all hip fractures. Intertrochanteric fracture usually occurs in patients with a history of falls or bone disease, and results from a low-energy mechanism such as a fall from standing [3]. Patients typically present with pain and difficulty walking.

Types of intertrochanteric fracture [4] and treatment [5] affect functional outcomes and mortality in patients with hip fracture. Patients with intertrochanteric fractures incur more blood loss than those with femoral neck fractures and have a higher rate of transfusion [6]. In addition, perioperative hemoglobin and hematocrit levels have implications for outcomes, as patients with hip fracture are usually frail and elderly and particularly prone to anemia and hypovolaemia [7–9]. Evidence suggests that total blood loss during hip fracture surgery may be much greater than that observed intraoperatively. One study showed that overall blood loss was 1473 mL greater than that observed intraoperatively in patients undergoing hip surgery [6], and another study [10] reported 277.2 ± 7.6 mL hidden blood loss in patients undergoing proximal femoral nail anti-rotation (PFNA) for intertrochanteric fractures. Hidden blood loss could aggravate functional outcomes and increase mortality in patients with hip fracture by lowering hemoglobin levels. Hidden blood loss should be minimized during surgery for intertrochanteric fracture.

Tranexamic acid (TXA) is a synthetic derivative of the amino acid lysine, with antifibrinolytic properties that competitively inhibit lysine-binding sites on plasminogen molecules. TXA has been used for hemostasis in orthopedic surgery [11–16]. Previous studies have shown that TXA reduced total blood loss and the need for transfusion in hip arthroplasty and hip fracture surgery [14, 15, 17]. However, the majority of these studies focused on the hemostatic effect of TXA on visible blood loss in hip fracture surgery, rather than on postoperative hidden blood loss [18]. One study showed that TXA decreased external blood loss by 30%, but not hidden blood loss, in total knee replacement [19]. Other reports in total knee arthroplasty show that TXA significantly reduced hidden blood loss and total blood loss [20–22], but there have been few studies investigating whether TXA can reduce hidden blood loss in surgery for intertrochanteric fractures [18, 23].

In this study, we hypothesized that TXA administration would lead to decreased postoperative hidden blood loss in patients with intertrochanteric fractures. The objective of this study was to investigate whether intravenous (i.v.) administration of 1 g TXA could reduce postoperative hidden blood loss in patients with intertrochanteric fractures.

## Methods

### Study population

This prospective study was a single-blinded randomized controlled clinical trial conducted at a single center. Patients with stable and unstable intertrochanteric fractures admitted to our institution through the emergency department between December 1, 2015 and July 5, 2016 were eligible for this study. Inclusion criteria were (1) patients with a definite history of trauma, fall or traffic accident; (2) patients suffering from hip pain, tenderness, dysfunction, local swelling, and vertical percussion pain in the area of the greater trochanter, with limited function in the injured limb; (3) patients with a confirmed diagnosis of intertrochanteric fracture and fracture classified according to AO type on X-ray or computed tomography (CT) [24]; and (4) patients eligible for intertrochanteric fracture surgery using the proximal femoral nail anti-rotation (PFNA) system (TianJin ZhengTian, XiaMen Double), as determined by the senior orthopedic surgeons at our institution.

Exclusion criteria were (1) patients with allergy to TXA; (2) patients with recent or ongoing thromboembolic events (deep venous thrombosis, pulmonary embolism, arterial thrombosis, or cerebral thrombosis stroke); (3) patients who were recently taking or who were taking anticoagulation therapy including vitamin K-antagonists, direct thrombin inhibitors, direct factor X-a inhibitors, and platelet aggregation inhibitors; (4) patients with disseminated intravascular coagulation or patients had hepatic or renal diseases with impairment of coagulation function; or (5) patients with a history of subarachnoid bleeding, malignancy, pathological fracture, or prior surgery on the injured hip.

This study was approved by the Ethics Committee of Xi’an Jiaotong University, and each patient provided written informed consent before surgery. This study was performed in line with the Declaration of Helsinki international ethical guidelines for studies involving human subjects [25].

### Intervention

Patients were randomized to a TXA group or a normal-saline (NS) group using a random number table. If patients were anemic (defined as hemoglobin <90 g/L) on admission they received an i.v. infusion of RBC. After anesthesia, but before surgery, patients in the TXA group received i.v. TXA 1 g (200 mL), and patients in the NS group received 200 mL of NS (i.v). A single orthopedic surgeon (LJL) performed surgery on all included patients. Patients were placed in supine position, the fractured bone fragments were identified by X-ray, and PFNA was performed.

### Outcome measurements

Patient demographic and clinical characteristics were recorded. Hemoglobin and hematocrit levels 1 day before surgery and on postoperative Day 1 and 3; duration of surgery; and visible blood loss collected with a sterile plastic foil, a funnel, and gauzes were measured. Complications associated with surgery—including hematoma, infection, deep vein thrombosis (examined by ultrasonography on day 3 post-operation), pulmonary embolism, myocardial infarction, ischemic cerebral infarction, respiratory infection, and renal failure—were also recorded.

Nadler’s formulae for blood volume and visible and hidden blood loss were applied after surgery: [26–28]

$$ \mathrm{Womenboodvolume}\left(\mathrm{L}\right)=\mathrm{height}{\left(\mathrm{m}\right)}^3\times 0.356+\mathrm{weight}\left(\mathrm{kg}\right)\times 0.033+0.183 $$

$$ \mathrm{Men}\;\mathrm{bloodvolume}\left(\mathrm{L}\right)=\mathrm{height}{\left(\mathrm{m}\right)}^3\times 0.356+\mathrm{weight}\left(\mathrm{kg}\right)\times 0.032+0.604 $$

$$ \mathrm{Total}\ \mathrm{RBC}\ \mathrm{loss}\left(\mathrm{L}\right)=\mathrm{blood}\  \mathrm{volume}\times \left({\mathrm{Hct}}_{Preop}\hbox{-} {\mathrm{Hct}}_{Postop}\right) $$

$$ \mathrm{Visible}\ \mathrm{RBC}\ \mathrm{loss}=\left(\mathrm{surgical}\  \mathrm{blood}\  \mathrm{loss}+\mathrm{postoperative}\  \mathrm{drainage}\right)\times \frac{\left({\mathrm{Hct}}_{Preop}+{\mathrm{Hct}}_{Postop}\right)}{2} $$

$$ \mathrm{Hidden}\kern0.5em \mathrm{RBC}\ \mathrm{loss}=\mathrm{total}\ \mathrm{RBC}\ \mathrm{loss}\hbox{-} \mathrm{visible}\ \mathrm{RBC}\ \mathrm{loss}+\mathrm{transfusion}\ \mathrm{RBC} $$

### Statistical analysis

Data were analyzed using SPSS v18.0 statistical software (SPSS Inc., Chicago, IL, USA). According to previous literature [13] and a power analysis, at least 72 patients were required for this study. Descriptive data are presented as mean ± SD. The chi-squared test or Student’s *t* test was used to compare demographic and clinical characteristics. A non-parametric test was used to evaluate ASA classification. *P* < 0.05 was considered statistically significant.

## Results

**Fig. 1:**
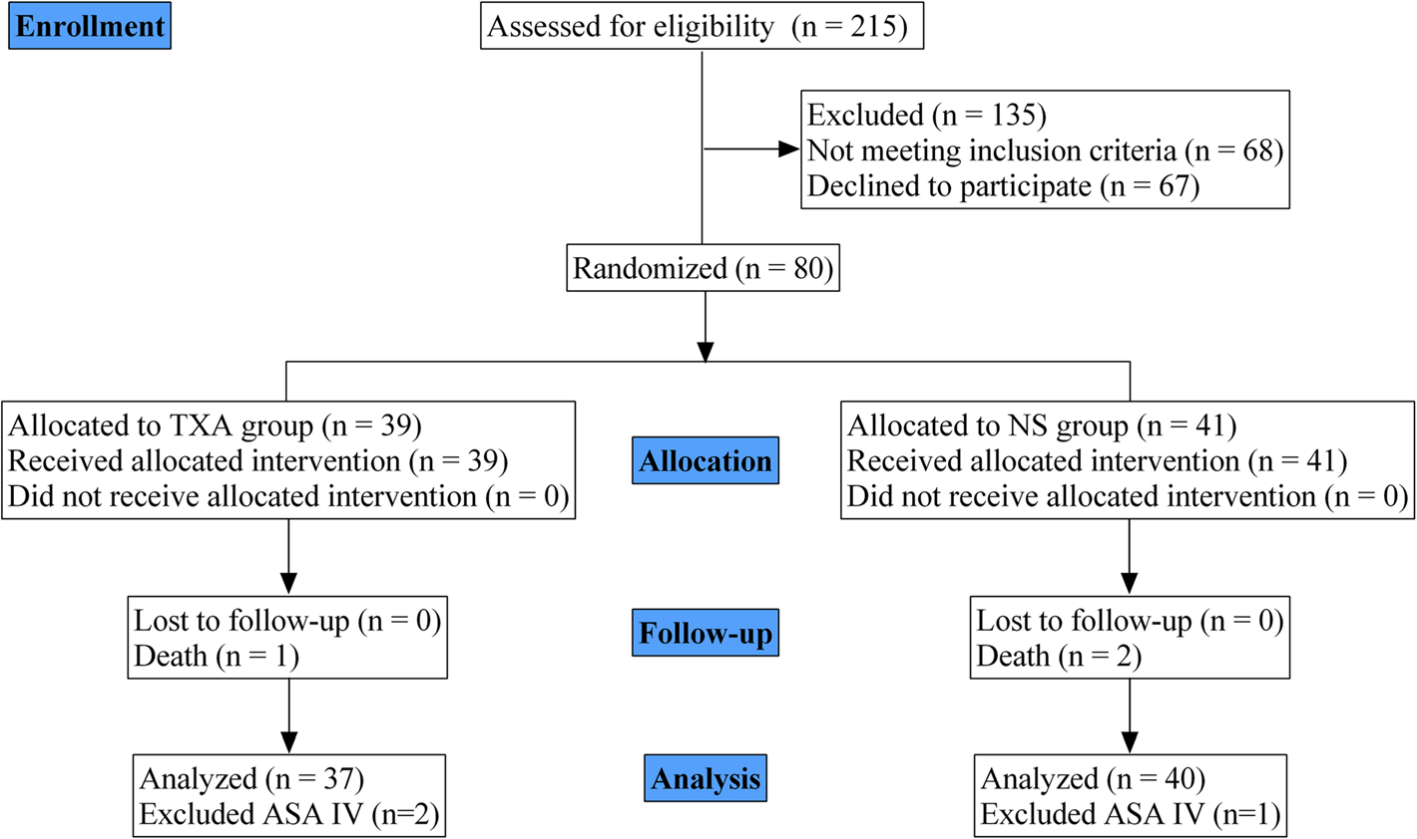
Flow chart of patient enrollment

Between December 2015 and July 2016, 215 patients with intertrochanteric fractures were admitted to our institution through the emergency department. Seventy-seven patients who met all of the inclusion criteria and none of the exclusion criteria were randomized to the TXA (*n* = 37) and NS groups (*n* = 40) (Fig. 1). Patients’ demographic and clinical characteristics were similar between the two groups as summarized in Table 1. Most patients (81.81%) were female aged 64 to 93 years. Upon admission, the hemoglobin level in 16 patients was <90 g/L; these patients received a total of 48.0 U of packed RBC by intravenous infusion. Four patients in the TXA group and four patients in the NS group each received 4 U packed RBC. Before surgery, hemoglobin level in most patients was between 91 and 137 g/L and hematocrit was between 27.6 and 34.6%.

**Table 1:** Demographic and clinical characteristics of the included patients

Variables	TXA study group (*n* = 37)	NS study group (*n* = 40)	*P* value
Female (%)	32 (82.05)	33 (80.49)	0.86^†^
Age (year)	77.80 ± 9.75	79.18 ± 6.50	0.54*
BMI	23.79 ± 2.18	23.27 ± 2.93	0.61*
Side (right/left)	18/19	21/19	0.74^†^
Preop. hemoglobin level (g/L)	109.50 ± 15.07	112.15 ± 13.31	0.51
Preop. hematocrit level (%)	32.63 ± 4.28	33.79 ± 3.83	0.31
Preop. transfusion (mL)	220.00 ± 354.80	158.00 ± 315.30	0.51
AO fracture classification			
31 A1	14	14	0.76^†^
31 A2	11	15	
31 A3	12	11	
ASA classification			
I	5	4	0.89^
II	16	18	
III	16	18	
IV	2	1	
Operative time (min)	81.90 ± 25.61	80.67 ± 29.44	0.88*
Hospital stay (days)	8.10 ± 1.74	9.03 ± 2.10	0.10*

*Two-sided student’s *t* test; ^†^chi-squared test; ^non-parametric test; *TXA* tranexamic acid, *NS* normal-saline, *BMI* body mass index

All surgeries were successful. Mean operative time and mean length of hospital stay were not significantly different between the TXA and NS groups. There was less surgical blood loss in the TXA group compared to the NS group (128.85 ± 123.18 vs. 98.30 ± 59.10; *P* = 0.31), but the difference was not significant. However, the postoperative transfusion rate was significantly lower in the TXA group compared to the NS group (TXA, 28.20% [2.0 U packed RBC in 10 patients, 4.0 U packed RBC in 1 person] vs. NS, 56.09% [2.0 U packed RBC in 18 patients, 4.0 U packed RBC in 5 patients]; *P* = 0.01). In the NS group, three patients >90 years of age received an intraoperative transfusion of 2 U packed RBC each.

As shown in Table 2, on postoperative day 1, both mean hematocrit and mean hemoglobin levels were not significantly different in the TXA group compared to the NS group. Postoperative drainage at postoperative day 2 was also not significantly different between the TXA and NS group (89.50 ± 44.77 mL vs. 89.73 ± 33.30 mL, *P* = 0.98). On postoperative day 3, mean hemoglobin and mean hematocrit levels were comparable. But the calculated hidden RBC loss (210.09 ± 202.14 mL vs. 359.35 ± 290.12 mL, *P* = 0.049) and total RBC loss (279.35 ± 209.11 mL vs. 417.89 ± 289.56 mL, *P* = 0.049) were significantly less in the TXA group compared to the NS group (Table 2).

**Table 2:** Comparison of postoperative clinical outcomes between the TXA group and NS group

Variables	TXA study group (*n* = 37)	NS study group (*n* = 40)	*P* value
Surgical blood loss (mL)	128.85 ± 123.18	98.30 ± 59.10	0.31
Postoperative day 2 drainage. (mL)	89.50 ± 44.77	89.73 ± 33.30	0.98*
Hemoglobin preop. (g/L)	109.50 ± 15.07	112.15 ± 13.31	0.51*
Hemoglobin postop. day 1 (g/L)	102.70 ± 13.18	115.27 ± 60.93	0.37*
Hemoglobin postop. day 3 (g/L)	101.05 ± 11.91	100.30 ± 12.54	0.83*
Hematocrit preop. (%)	32.63 ± 4.28	33.79 ± 3.83	0.31*
Hematocrit postop. day 1 (%)	30.53 ± 3.81	31.15 ± 3.80	0.56*
Hematocrit postop. day 3(%)	30.23 ± 3.23	29.34 ± 5.87	0.54
Estimated visible RBC loss day 3 (mL)	69.26 ± 44.77	58.55 ± 22.10	0.33*
Transfusion rate (%)	11/39 (28.20%)	23/41 (56.09%)	0.01^†^
Transfusion units (U)	24	56	
Estimated total RBC loss day 3 (mL)	279.35 ± 209.11	417.89 ± 289.56	0.049*
Estimated hidden RBC loss day 3 (mL)	210.09 ± 202.14	359.35 ± 290.12	0.049*

*Two-sided Student’s *t* test; †chi-squared test; *TXA* tranexamic acid, *NS* normal-saline

There were no systematic complications related to TXA administration. The incidence of adverse events in the TXA and NS group were not significantly different (Table 3). Patients with hematoma and infection at the surgical site were treated conservatively, but one patient required surgical debridement.

**Table 3:** Postoperative complications in the TXA group and NS group

Complications	TXA study group (*n* = 37)	NS study group (*n* = 40)	*P* value*
Surgical site			
Hematoma	1	3	0.34
Infection	1	2	0.60
Medical			
Deep vein thrombosis	2	1	0.51
Pulmonary embolism	1	1	0.96
Myocardial infarction	0	0	
Ischemic cerebral infarction	0	0	
Respiratory infection	3	5	0.53
Renal failure	0	1	1.00

*Chi-squared test; *TXA* tranexamic acid, *NS* normal-saline

All patients were followed up for 30 days after surgery. Three patients were lost to follow-up due to death (2 of pulmonary embolism and 1 of renal failure). Deep vein thrombosis resolved spontaneously.

## Discussion

Hip fracture surgery may result in substantial blood loss in elderly and frail patients, exposing them to postoperative anemia, which could negatively impact clinical outcomes and mortality. Previous studies have shown that TXA reduced hidden blood loss associated with total knee arthroplasty [22]. However, it is not clear whether TXA decreases hidden blood loss in patients undergoing PFNA for intertrochanteric fractures. Our results showed that both postoperative hidden blood loss and total blood loss were significantly reduced in patients with intertrochanteric fractures treated with TXA, suggesting TXA administration is an efficacious approach to reducing blood loss in patients undergoing PFNA for intertrochanteric fractures.

Our results showed that compared to NS, TXA administration reduced postoperative RBC loss to 279.35 mL and hidden blood loss to 700.3 mL (assuming 30% hematocrit) in patients with intertrochanteric fractures. These results are consistent with previous studies reporting on total knee arthroplasty [12], periacetabular osteotomy [11], extracapsular fracture of the hip [13], and total shoulder arthroplasty [16]. Our data also suggests that TXA administration has the potential to decrease the number of orthopedic patients requiring transfusion; administration of 1 g of TXA decreased the transfusion rate from 56.09 to 28.20%. This would contribute to a substantial reduction in healthcare costs and resource utilization for these patients.

TXA competitively blocks a lysine-binding site of plasminogen and thereby inhibits its conversion to the active enzyme plasmin. Plasmin binds to fibrinogen or fibrin structures and promotes fibrinolysis [29]. Additional evidence suggests that plasmin is pro-inflammatory [30]. Currently, it is debatable whether TXA benefits trauma patients through reversal of fibrinolysis or modulating the inflammatory response [31, 32].

In this study, we chose to administer 1 g of TXA or NS i.v. after anesthetization and before surgery. We used a low dose and systemic administration, as reported by Wingerter et al. [33]. In contrast, Drakos et al. [23] administered 3 g of TXA around the fracture site at the end of the surgical procedure in patients undergoing surgery for intertrochanteric fracture, and Tengberg et al. [13] administered 1 g of TXA as an intravenous bolus prior to surgery followed by a postoperative 24-h infusion of 3 g TXA in 1 L of isotonic saline in patients undergoing surgery for extracapsular hip fracture.

The most common complication associated with TXA administration is ischemic cerebral infarction at postoperative 1 month after operation. However, there was no ischemic cerebral infarction in our study and there were no significant differences in the incidence of adverse events between the TXA and NS groups. TXA is a synthetic derivative of the amino acid lysine and may, therefore, be associated with thrombotic complications; however, recent large studies and meta-analyses have not consistently reported an increased risk for thrombosis [34–36]. The overall complication rate in our study was comparable with previous reports [13, 23].

To our knowledge, this is the first randomized controlled trial of TXA vs. NS for postoperative hidden blood loss in patients undergoing PFNA for intertrochanteric fractures. Only patients eligible for PFNA were included; therefore, the demographic and clinical characteristics of the TXA and NS groups were comparable.

The study has some limitations. First, the sample size is relatively small. Second, this study was not double-blind. Third, the fracture type was limited to intertrochanteric fractures. Future studies using larger sample sizes and a variety of fracture types are warranted to confirm our findings.

## Conclusion

This study demonstrated that TXA could effectively reduce postoperative hidden blood loss in patients undergoing PFNA for intertrochanteric fractures and may decrease the number of patients needing transfusion.

## Abbreviations

CT: Computed tomography
Hb: Hemoglobin
Hct: Hematocrit
NS: Normal saline
PFNA: Proximal femoral nail anti-rotation
RBC: Red blood cell
TXA: Tranexamic acid
